# Impact of the terminal end-group on the electrical conductance in alkane linear chains†

**DOI:** 10.1039/d3ra00019b

**Published:** 2023-02-17

**Authors:** Abdullah Alshehab, Ali K. Ismael

**Affiliations:** a Physics Department, College of Science, King Faisal University Al Ahsa Saudi Arabia; b Department of Physics, Lancaster University Lancaster LA1 4YB UK k.ismael@lancaster.ac.uk

## Abstract

This research presents comprehensive theoretical investigations of a series of alkane-based chains using four different terminal end groups including amine –NH_2_, thiomethyl –SMe, thiol –SH and direct carbon contact –C. It is widely known that the electrical conductance of single molecules can be tuned and boosted by chemically varying their terminal groups to metal electrodes. Here, we demonstrate how different terminal groups affect alkane molecules' electrical conductance. In general, alkane chain conductance decreases exponentially with length, regardless of the anchor group types. In these simulations the molecular length varies from 3 to 8 –CH_2_ units, with 4 different linker groups; these simulations suggest that the conductances follow the order *G*_C_ > *G*_SH_ > *G*_SMe_ > *G*_NH_2__. The DFT prediction order of the 4 anchors is well supported by STM measurements. This work demonstrates an excellent correlation between our simulations and experimental measurements, namely: the percent difference Δ*G*, exponential decay slopes, *A* constants and *β* factors at different molecular alkane chain lengths.

## Abstract 2

This research presents comprehensive theoretical investigations of a series of alkane-based chains using four different terminal end groups including amine –NH_2_, thiomethyl –SMe, thiol –SH and direct carbon contact –C.

## Graphical abstract

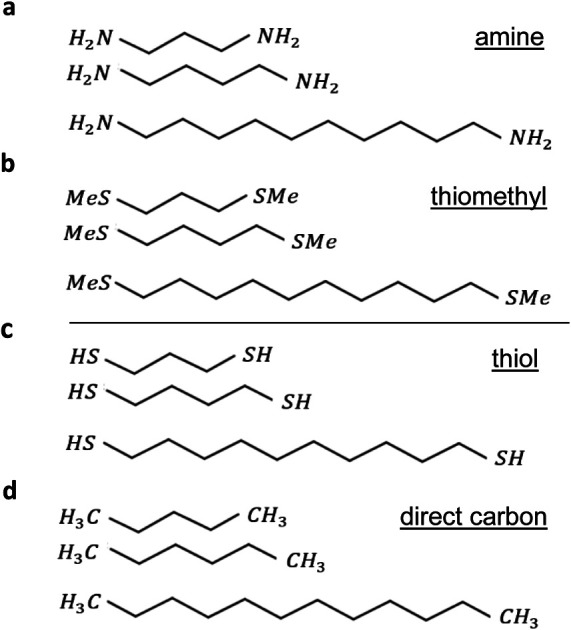

## 1 Introduction

Molecular scale electronics relies on an understanding of the transport characteristics of molecules bonded to metal electrodes. Molecular properties, including their length, conformation, gap between the highest occupied and lowest unoccupied molecular orbitals, and the alignment of this gap with the metal Fermi level, play a significant role in these characteristics.^1–8^ Moreover, the electrical conductance and Seebeck coefficient of single molecules can be controlled in a deterministic manner by chemically varying their anchor groups to external electrodes and this research focuses on this parameter.^9–16^

In this study, we look to demonstrate that the nature or type of anchor groups used to bind molecules to metal electrodes also affects transport through single-molecule junctions.^17–20^ This research describes the effects of anchoring groups on the conductance of single molecules using *n*-alkane single chains as a model system.^21^ In the integrated circuit (IC) industry, multi-energy molecules are ideal candidates for further miniaturization of switches, logic gates, sensors, memories and other electronic devices.^22–25^ Initially, we began by investigating *G* of linear alkane chains as a first step toward understanding *G* of alkane molecules by employing 4 different anchor groups involving: amine (–NH_2_), thiomethyl (–SMe), thiol (–SH) and direct carbon contact (–C). This study aims to demonstrate the effect of using 4 different terminal groups on the *G* of alkane chains and benchmark our results against available published experimental data.

## 2 Methods

To begin, we started by modelling terminal groups–Au binding, and then relaxed each compound in the presence of fixed leads. Using the density functional (DFT) code SIESTA^26^ (for more detail see geometry of isolated alkane in the ESI†) the optimum geometries of isolated alkane linear chains were obtained by relaxing the molecules until all forces on the atoms were less than 0.01 eV Å^−1^, and 1 *k*-point (see ESI Fig. 1†). We used a double-zeta plus polarization orbital basis set, norm-conserving pseudopotentials, the local density approximation (LDA) exchange correlation functional, and to define the real space grid, an energy cutoff of 250 Rydbergs. We also computed results using GGA and found that the resulting transmission functions were comparable^27,28^ with those obtained using LDA. To simulate the likely contact configuration during a break-junction experiment, we employed leads constructed from 6 layers of Au (111), each containing 30 gold atoms and further terminated with a pyramid of gold atoms. After relaxing each molecular junction with lengths varying from *n* = 3 to *n* = 10, we calculated the electrical conductance using the Gollum quantum transport code^29^ (for more detail see Section 4 of the ESI†).

**Fig. 1:**
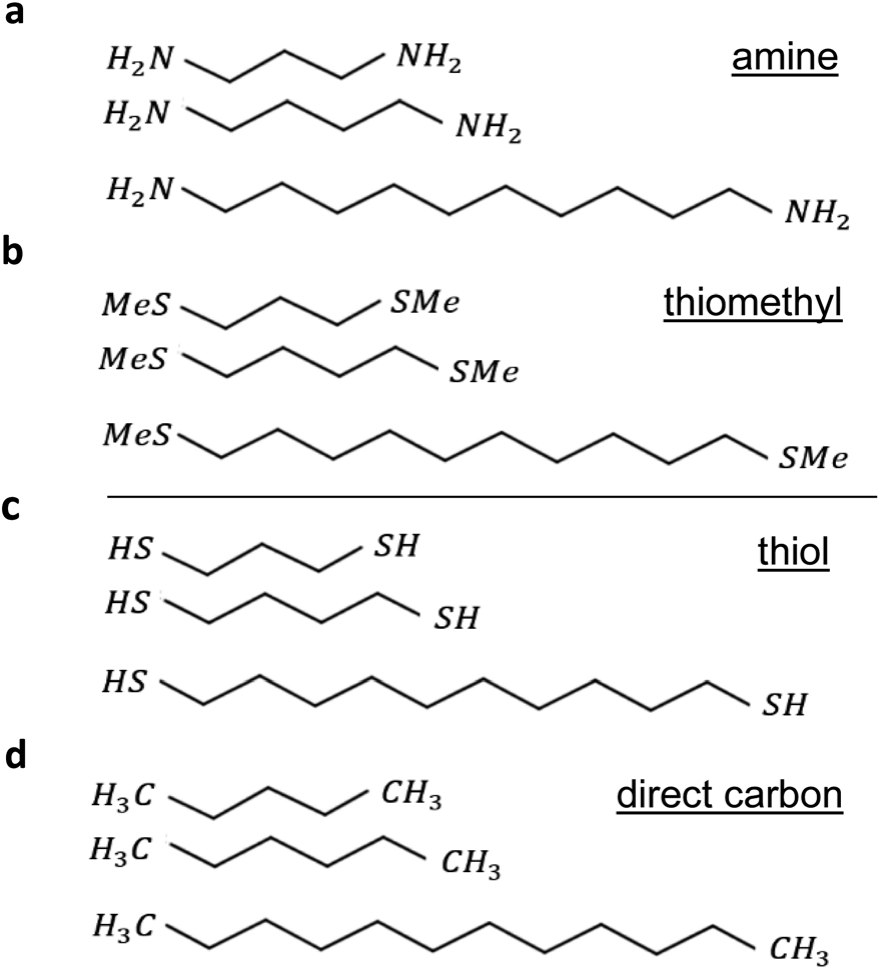
Examples of alkane linear chain derivatives with four different terminal groups: (a) amine *n* = 3, 4, … 10 linear chains. (b) Thiomethyl *n* = 3, 4, … 10 linear chains. (c) Thiol *n* = 3, 4, … 10 linear chains. (d) Direct carbon *n* = 3, 4, … 10 linear chains (for clarity only 3 chains out of 8 are shown for each terminal group).

The current research explores 32 molecules with 4 different anchor groups as illustrated in Fig. 1 below. Fig. 1a below, displays 8 linear chains that terminated with amine. Panel (b), shows another 8 chains, however, they terminated with thiomethyl anchors. Similarly, panels (c and d), involve 8 chains each and terminated with thiol and direct carbon respectively (note: for clarity only 3 chains are shown in each panel).

All the studied linear chains are coupled to gold electrodes through the 4 terminal end groups and as follows: the first group *via* amine anchor (Au–NH_2_), second group *via* thiomethyl anchor groups (Au–SMe), third thiol anchor (Au–S), and the last group *via* the covalent bond to form a carbon direct contact (Au–C). Examples of linear chains with four different anchor groups that coupled to gold electrodes are shown in ESI Fig. 3.†

To aid the discussion of the 4 group conductances, we assign an integer *n* to each chain (see Fig. 1), such that chains of equal *n* have comparable lengths. For Au–NH_2_, Au–SMe, and Au–S terminated chains, *n* is defined to equal the number of methyl units along the length of the chain between the two anchors (*i.e.*, carbons, nitrogens and sulfurs). For the Au–C linked chains, where the terminal –CH_2_ units are regarded as anchor groups, *n* is defined to be two less than the number of –CH_2_ units.

In continuation, we will examine 32 linear alkane chains. Gold electrodes are bound to these terminal groups by different binding energies. Thiol anchor has the highest binding energy at −2 eV, followed by direct carbon anchor at −1.8 eV, thiomethyl anchor at −0.3 eV, and amine anchor at −0.15 eV. Linear molecular chains have been well-studied theoretically^30,31^ and experimentally^32–34^ and found that their *G* is found to decay exponentially with length, as *G*(*n*) ≈ exp − *βn*, where *n* is number of methylene –CH_2_ units and *β* is decay constant per –CH_2_ unit. According to previous experiments, the decay constant *β* is approximately 0.9 per methylene, with a fluctuation of 10% depending on the type of terminal group.^35–38^

## 3 Results and discussion

The transport properties of 32 linear alkane junctions involving 4 different anchor groups were modelled using a combination of density functional theory (DFT) and quantum transport theory. To have a better understanding of electronic properties, the frontier orbital of studied molecules: highest occupied molecular orbitals (HOMO) and lowest unoccupied orbitals (LUMO) along with their energies are investigated as shown in Tables. S1–S12 of the ESI.†

For the terminal Au–NH_2_, the covalent bond distance is found to be 3 Å. For SMe-terminal the Au–S distance is slightly bigger at 2.8 Å, for thiol-terminal the Au–S distance is 2.4 Å and for direct carbon-terminal the Au–C distance is 2.3 Å as illustrated in ESI Fig. 2,† (for more detail see the binding energy simulations in the ESI).

As a first step, we investigated transport through these alkane derivatives in Au–Au junctions. Each anchor group illustrates a unique type of transport, in other words HOMO, LUMO or med-gap transport. For example, amine anchor (–NH), exhibits mid-gap transport (halfway between the HOMO and LUMO resonances), as shown in ESI Fig. 4,† whereas thiomethyl anchor shows LUMO-dominated (Lowest Unoccupied Molecular Orbital) transport hinting that the –SMe moves the LUMO closest to the Fermi energy (see ESI Fig. 5†). In contrast, thiol anchor exhibits HOMO-dominated (Highest Occupied Molecular Orbital) transport indicating that the –SH moves the HOMO closer to the Fermi energy (see ESI Fig. 6†). Similarly, for the direct carbon bound as shown in ESI Fig. 7,† (for more detail see Section 5 of the ESI).

**Fig. 2:**
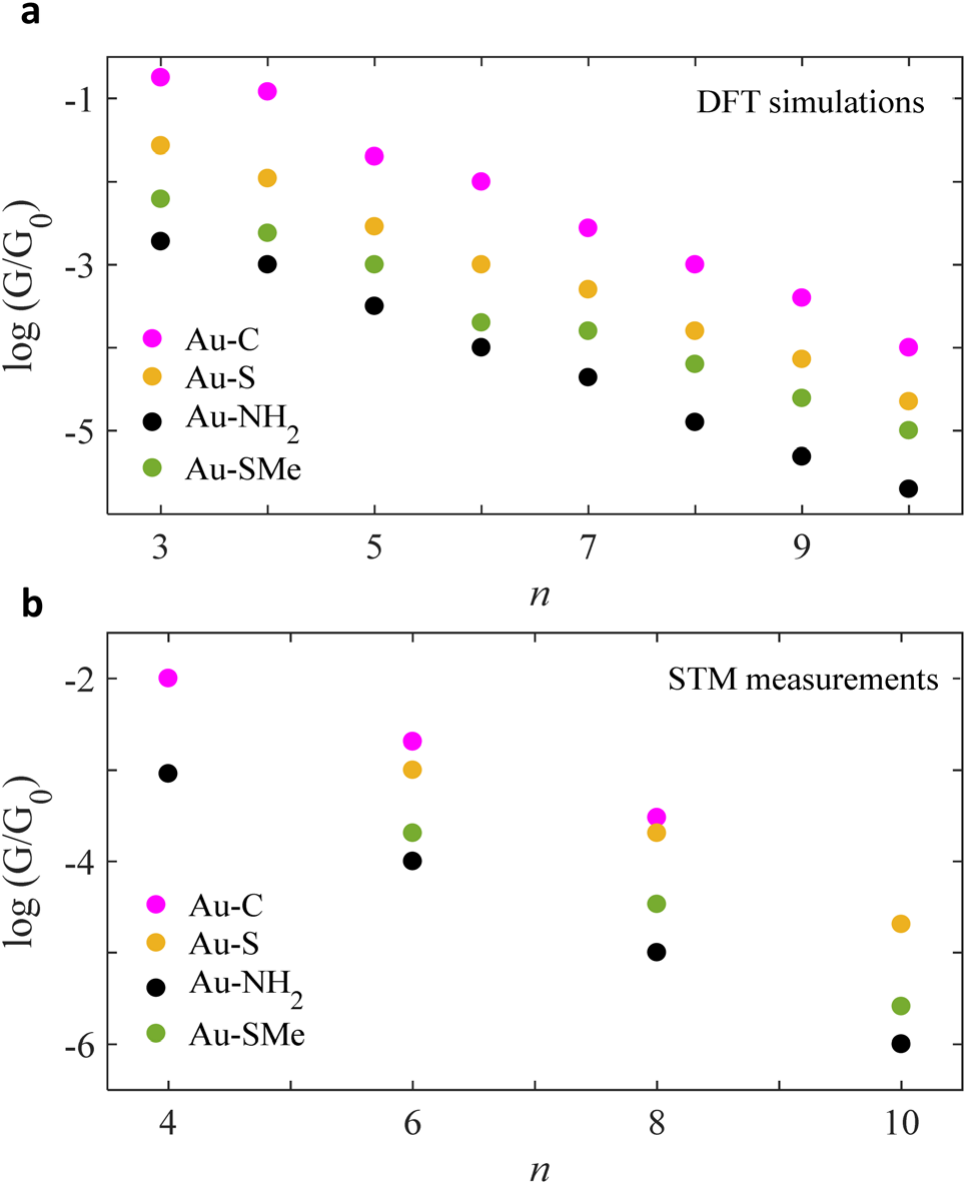
Chain-length conductance dependence of Au|alkane linear chain|Au junctions for four different terminal groups. Logarithmic conductance of DFT and STM (a and b respectively), as a function of the chain length of four different terminal groups involving direct carbon contact, thiol, amine and thiomethyl (Au–C: purple circles, Au–S: orange circles, Au–NH_2_: black circles, Au–SMe: green circles). Note: for more information about the experimental measurements see ref. 21, 31, 39 and 40.

ESI Fig. 4–7† show the conductance *G* for linear alkane chains as a function of length with four different terminal groups including: amine, thiomethyl, thiol and direct contact Au–C. These findings prove that *G* is sensitive to the terminal group and demonstrates that the conductance is highest for the covalent bond Au–C and lowest for the amine (–NH_2_) terminal group as shown in Fig. 2. Fig. 2a, displays the theoretical simulations of the electrical conductance for the 4 studied anchors. Here, the length of chains varies from *n* = 3 to *n* = 10. It demonstrates the conductance varies from approximately −2.2 *G*_0_ to −5 *G*_0_ when the molecular length increases from 3 to 10 –CH_2_ units. This modulation is more than double when we compare the amine against the covalent bond Au–C anchor (specifically *n* < 6). Ideally, in molecular electronics devices it is essential to have both high and low conductance (on/off). Thus, the covalent and amine anchors secure the high and low required conductance. Fig. 2b, shows the experimental measurements for the same anchor groups, however, not the full length is measured (*n* = 4, 6 and 8). By comparing the two panels of Fig. 2, one could notice there is an excellent similitude between the DFT simulations and the STM measurements.^21,31,39,40^

Within the alkane linear chain series, the conductance of different anchors follows the order of direct carbon bound, thiol, thiomethyl and amine (Au–C > Au–S > Au–SMe > Au–NH_2_), which is in an excellent agreement with the experimentally measured trend. It should be noted that terminal groups of a strong binding energy to Au surface yield high *G* compared to the weak ones. For example, B.E of (Au–C and Au–S) > (Au–SMe and Au–NH_2_), for more detail see Section 3 in the ESI.†

To have insight view about the comparison between our simulations and measurements, we constructed Tables 1 and 2, to compare point by point (*i.e.*, *n* × *n* of similar length), and show the difference between DFT and STM conductances Δ*G*1
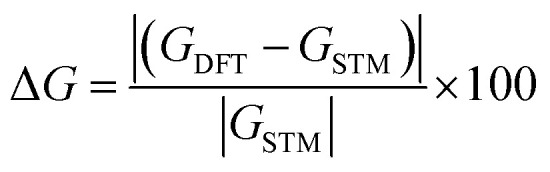
where *G*_DFT_ and *G*_STM_: the calculated and measured conductances respectively.

**Table 1:** Percent difference Δ*G* between experimental measurements (STM), and theoretical calculations (DFT), of different molecular length (*n*), for amine and thiomethyl

Anchor	*n*	DFT log(*G*/*G*_0_)	STM log(*G*/*G*_0_)	Δ*G* (%)
	3	−2.7	—	—
–NH_2_	4	−3.0	−3.04 (ref. 21)	1.3
	5	−3.5	—	—
	6	−4.0	−4.0 (ref. 21)	0.0
	7	−4.3	—	—
	8	−4.9	−5.0 (ref. 21)	2.0
	9	−5.3	—	—
	10	−5.7	−6.0 (ref. 21)	5.0
–SMe	3	−2.2	—	—
	4	−2.6	—	—
	5	−3.0	—	—
	6	−3.7	−3.69 (ref. 31)	0.27
	7	−3.8	—	—
	8	−4.2	−4.47 (ref. 31)	6.04
	9	−4.6	—	—
	10	−5.0	−5.59 (ref. 31)	10.6

**Table 2:** Percent difference Δ*G* between experimental measurements (STM), and theoretical calculations (DFT), of different molecular length (*n*), for thiol and direct carbon bound

Anchor	*n*	DFT log(*G*/*G*_0_)	STM log(*G*/*G*_0_)	Δ*G* (%)
–SH	3	−1.5	—	—
	4	−1.9	—	—
	5	−2.5	—	—
	6	−3.0	−3.0 (ref. 21)	0.0
			−3.0 (ref. 40)	0.0
	7	−3.3	—	—
	8	−3.8	−3.69 (ref. 21)	2.98
	9	−4.1	−4.00 (ref. 40)	2.50
	10	−4.65	−4.69 (ref. 21)	0.85
			−4.50 (ref. 40)	3.30
–C	3	−0.7	—	—
	4	−0.92	−2.0 (ref. 39)	54
	5	−1.7	—	—
	6	−2.0	−2.69 (ref. 39)	25.6
	7	−2.5	—	—
	8	−3.0	−3.5 (ref. 39)	14.3
	9	−3.4	—	—
	10	−4.0	—	—

The conductance difference Δ*G* calculations (Tables 1 and 2), illustrate how successful our simulation is in predicting the electric conductance through alkane linear chains. It demonstrates the difference Δ*G* to be less than 3% for thiol derivatives, followed by 5% for amine then 10% for methyl sulphides and up to 50% for a single value for direct carbon derivatives as show in Tables 1 and 2.

Again, looking at Fig. 2 panels, it is noticeable that the slopes of the experimental measurements are in good agreement with our theoretical results and the actual values are summarised in the first and second columns of Table 3. This study, also calculates the exponentiation decay of each terminal end group as the conductance *G* was observed to decay exponentially with length (*n*) for both theory and experiment curves in Fig. 2.2
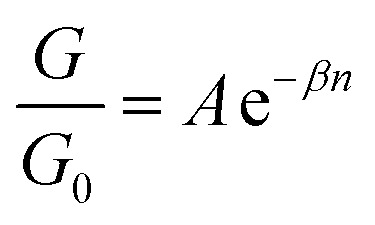
where *n* is number of methylene –CH_2_ units, *β* is decay constant per –CH_2_ unit and *A* is a constant.

**Table 3:** Comparison of some experimental and theoretical parameters including: slopes, *β* factors and the constant *A*, for the studied terminal groups. Note: for more information about the experimental measurements see ref. 21, 31, 39 and 40

Anchor	Slope _DFT_	Slope _STM_	*β*_DFT_	*β*_STM_	*A*_DFT_	*A*_STM_
–NH_2_	−0.44	−0.49 (ref. 21)	0.30–0.50	0.48–0.50 (ref. 21)	0.05	0.08 (ref. 21)
–SMe	−0.40	−0.48 (ref. 31)	0.20–0.80	0.40–0.56 (ref. 31)	0.18	0.16 (ref. 31)
–SH	−0.44	−0.42 (ref. 21)	0.30–0.60	0.34–0.50 (ref. 21)	0.50	0.40 (ref. 21)
				0.36–0.54 (ref. 40)		
–C	−0.47	−0.38 (ref. 39)	0.20–0.80	0.35–0.40 (ref. 39)	5.80	0.35 (ref. 39)

Table 3, shows a good agreement between *β*_DFT_ and *β*_DFT_, for a wide range of –CH_2_ unit and for all anchors. This demonstrates that *β* factors vary slightly from one anchor to another. All in all, one can surmise that *β* factor range from 0.2 to 0.8 per –CH_2_ unit for the 4 anchors. *β* represents the efficiency of electron transport along alkane chains and is regarded as an important parameter.^21^

On the other hand, *A* constant simply represents *Y*-intercept values which could be used to calculate the distance between two desired lines by taking the difference between their constants (see Fig. 2). Again, one could note likeness in the results between *A*_DFT_ and *A*_STM_, specifically for the first anchors (*i.e.*, –NH_2_, –SMe and –SH).

## 4 Conclusions

In summary, through a comprehensive study, we have demonstrated that the electrical performance of alkane-based molecular chains can be systematically tuned by varying the terminal end groups, coupling the alkane derivatives to metal electrodes. The electric conductance of a series of alkane linear chains of 4 different anchors have investigated and benchmarked against the measured conductance of similar alkane chains.

This study demonstrates that the conductance varies from one anchor to another and the order is *G*_C_ > *G*_SH_ > *G*_SMe_ > *G*_NH_2__. It also shows their logarithmic conductances decrease when the length (*n*), of chains increases. An excellent agreement was found between our simulations and experimental measurements through several parameters including the percent difference Δ*G*, exponential decay slopes, *A* constants and *β* factors at different molecular alkane chain lengths. Moreover, this research opens new ideas for designing electronic devices based on using different terminal end groups with potential practical applications.

## Author contributions

A. K. I. originally conceived the concept, calculations were carried out by A. A. Both authors have given approval to the final version of the manuscript. Both authors provided essential contributions to interpreting the data reported in this manuscript. A. K. I. coordinated the writing of the manuscript with input from A. A.

## Conflicts of interest

There are no conflicts to declare.

## Supplementary Material

RA-013-D3RA00019B-s001
