# Supplementary material for: Impact of the terminal end-group on the electrical conductance in alkane linear chains
Source: RSC Adv. 2023 Feb 17;13(9):5869–73. doi: 10.1039/d3ra00019b (PMC9936266; doi:10.1039/d3ra00019b)
Supplement: RA-013-D3RA00019B-s001 [file RA-013-D3RA00019B-s001.pdf]

Electronic Supplementary Material (ESI) for RSC Advances.
This journal is © The Royal Society of Chemistry 2023

Supporting Information

Impact of terminal end-group on the electrical conductance in alkane linear chains

Abdullah Alshehab [a] and Ali K. Ismael[a]*

[a.] Physics Department, Lancaster University, Lancaster, LA1 4YB, UK.

Table of contents

1. Theoretical details 2

1.1 Geometry of isolated alkane linear molecules 2

2. Frontier orbitals of the molecules 3

3. Binding energy of four terminal groups on gold 10

4. Optimised DFT Structures of Compounds in their Junctions 11

5. Conductance comparison between linear chains of different terminal groups 12

References 14

1. Theoretical details

1.1 Geometry of isolated alkane linear molecules

The DFT code (SIESTA)[1-2] was used to obtain fully relaxed geometries of the isolated alkane linear chains as shown in Supplementary Figure 1. In this work, we choose four different alkane linear chains based on their terminal groups. Supplementary Figure1 shows some examples of alkane chains with four different ancho groups. Group-a is terminated with amine while group-b, c and d are terminated with thiomethyl, thiol and direct carbon groups respectively. The length of each linear chain varies from n= 3 to 10 methylene (n= CH_2) units, as shown in Supplementary Figure1.

Here, we present the fully relaxed isolated conformations of twelve alkane linear chains. We started by three CH_2 units, then we increased the length by adding one CH_2 unit each time until n=10.

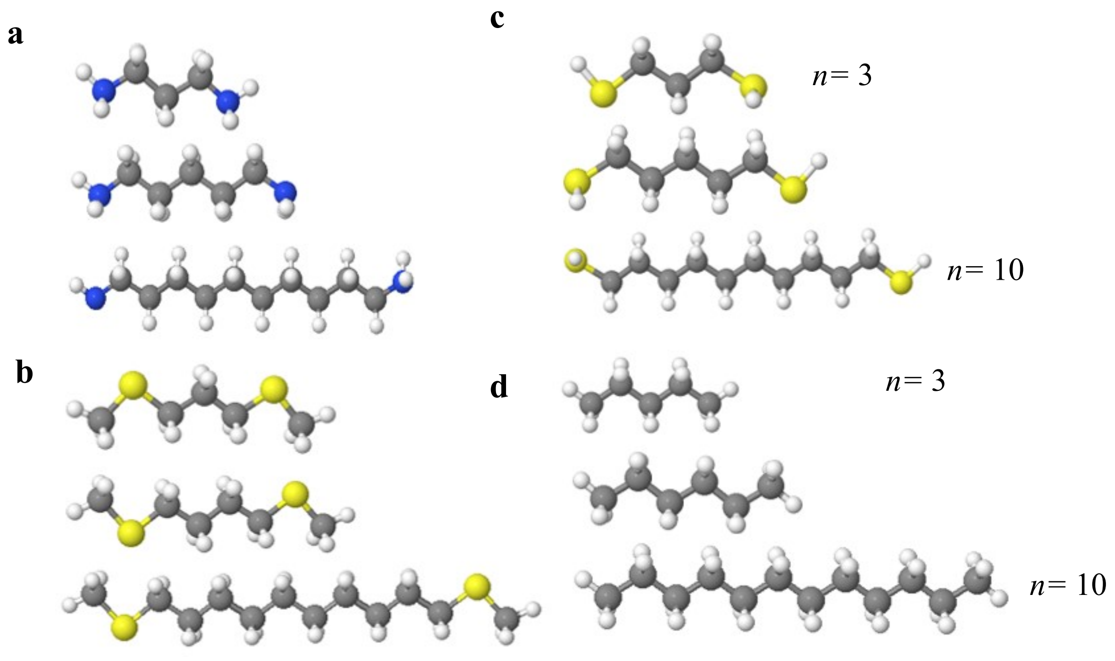

Supplementary Figure 1. Alkane linear chains: Fully relaxed isolated molecules, of different length 3-10 carbon atoms with four different terminal groups: (a): Amine n =3, 4, …. 10 linear chains. (b): Thiomethyl n =3, 4, …. 10 linear chains. (c): Thiol n =3, 4, …. 10 linear chains. (d) Direct carbon n =3, 4, …. 10 linear chains (for clarity only 3 chains out of 8 are shown for each terminal group).

2. Frontier orbitals of the molecules

To gain a deeper insight into the electronic properties of these alkane chain structures (see Supplementary Figure 1). The gas-phase electronic structures of all alkane chains were investigated to explore the distribution and composition of the frontier molecular orbitals. Plots of the frontier orbitals for the studied groups **a-d,** are given in Tables S1-S10. The highest occupied molecular orbitals (HOMO), lowest unoccupied orbitals (LUMO), HOMO-1 and LUMO+1 along with their energies are calculated. By comparing the topology of the HOMO and LUMO orbitals of 32 alkane molecules, one could notice that the HOMOs and LUMOs are extended across the backbone for each molecule, which suggests that they act as electron-electron channels.

Table S1. Comparison between the frontier molecular orbitals of alkane chain, including *n* =3, 4 and 5 linear chains in the gas phase (amine terminal group).

**Chains**	**3**	**4**	**5**
**Mol.**<br>**E_F (eV)**	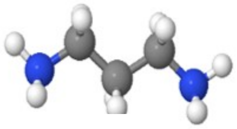<br>-1.1	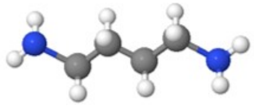<br>-1.0	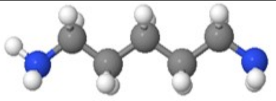<br>-1.03
**HOMO**<br>**eV**	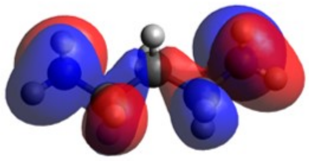<br>-4.5	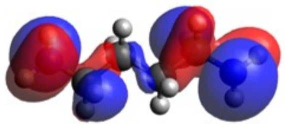<br>-4.6	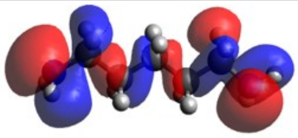<br>-4.5
**LUMO**<br>**eV**	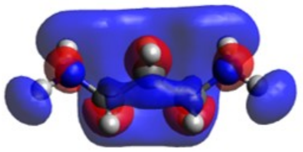<br>-2.1	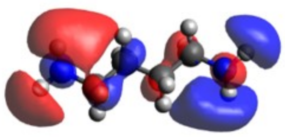<br>-2.1	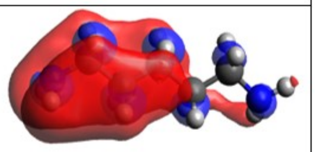<br>-2.0

Table S2. Comparison between the frontier molecular orbitals of alkane chain, including *n* =6, 7 and 8 linear chains in the gas phase (amine terminal group).

Chains	6	7	8
Mol.<br><br>E_F (eV)	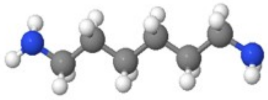<br>-2.4	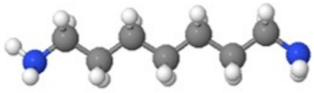<br>-2.3	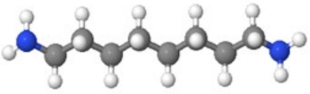<br>-2.3
HOMO<br><br>eV	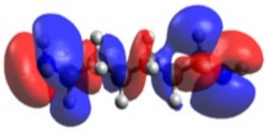<br>-4.63	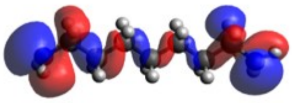<br>-4.7	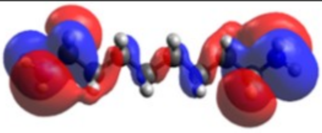<br>-4.66
LUMO<br><br>eV	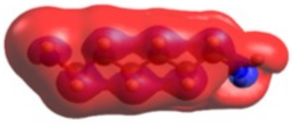<br>-1.9	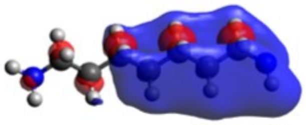<br>-1.9	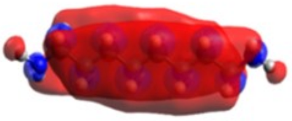<br>-2.0

Table S3. Comparison between the frontier molecular orbitals of alkane chain, including *n* =9 and 10 linear chains in the gas phase (amine terminal group).

Chains	9	10
Mol.<br><br>E_F (eV)	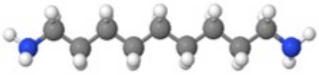<br>-2.1	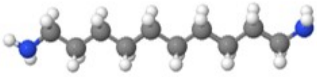<br>-2.0
HOMO<br><br>eV	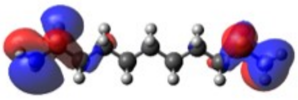<br>-4.7	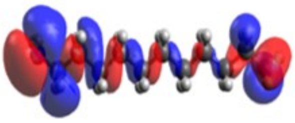<br>-4.65
LUMO<br><br>eV	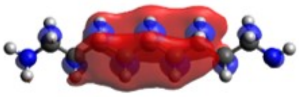<br>-2.0	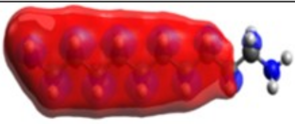<br>-1.96

Table S4. Comparison between the frontier molecular orbitals of alkane chain, including *n* =3, 4 and 5 linear chains in the gas phase (thiomethyl terminal group).

Chains	3	4	5
Mol.<br><br>$E_{F\,(eV)}$	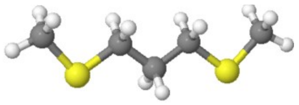<br>-0.80	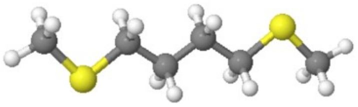<br>-2.87	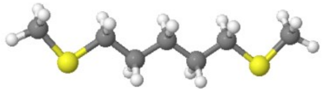<br>-2.58
HOMO<br><br>eV	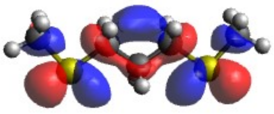<br>-0.18	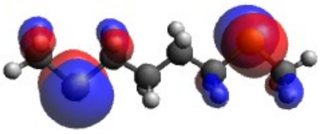<br>-4.17	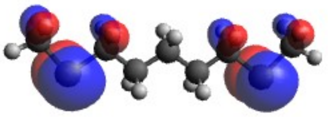<br>-4.14
LUMO<br><br>eV	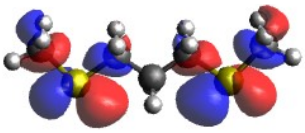<br>-1.34	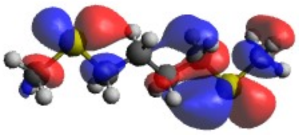<br>-1.13	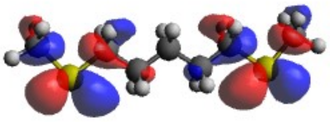<br>-1.10

Table S5. Comparison between the frontier molecular orbitals of alkane chain, including *n* =6, 7 and 8 linear chains in the gas phase (thiomethyl terminal group).

Chains	6	7	8
Mol.<br><br>$E_{F(eV)}$	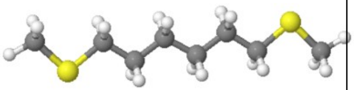<br>-1.93	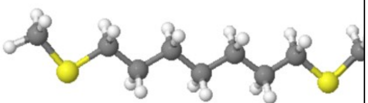<br>-1.54	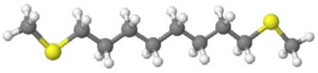<br>-1.30
HOM<br>O<br><br>eV	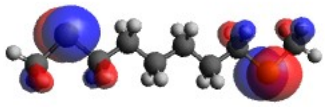<br>-4.16	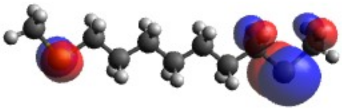<br>-4.14	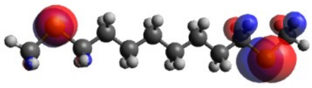<br>-4.13
LUMO<br><br>eV	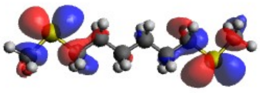<br>-1.02	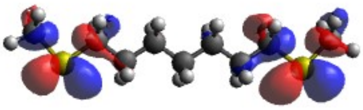<br>-1.18	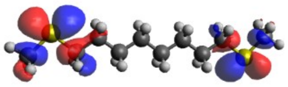<br>-1.19

Table S6. Comparison between the frontier molecular orbitals of alkane chain, including *n* =9 and 10 linear chains in the gas phase (thiomethyl terminal group).

**Chains**	**9**	**10**
**Mol.**<br>**$E_{F(eV)}$**	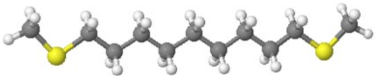<br>-1.11	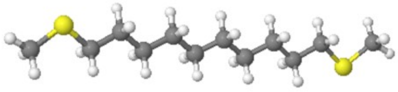<br>-0.84
**HOMO**<br>**eV**	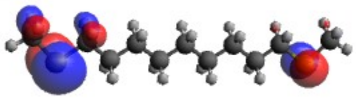<br>-4.13	-<br>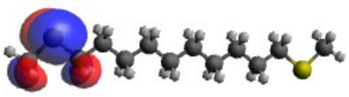<br>4.12
**LUMO**<br>**eV**	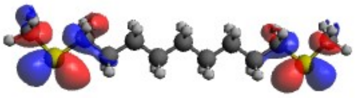<br>-1.29	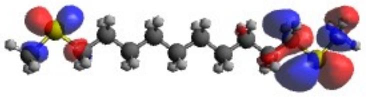<br>-1.20

Table S7. Comparison between the frontier molecular orbitals of alkane chain, including *n* =3, 4 and 5 linear chains in the gas phase (thiol terminal group).

**Chains**	**3**	4	**5**
**Mol.**<br><br>**E_F (eV)**	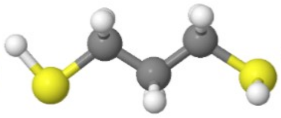<br>-0.49	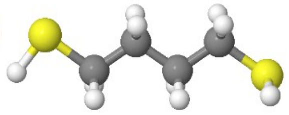<br>-3.3	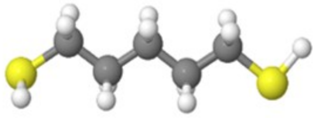<br>-2.8
**HOMO**<br><br>**eV**	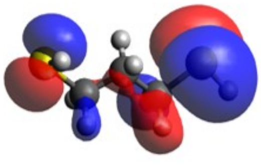<br>-4.8	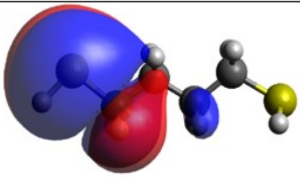<br>-4.80	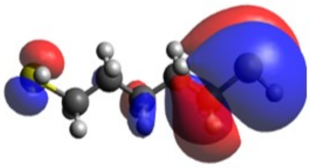<br>-4.7
**LUMO**<br><br>**eV**	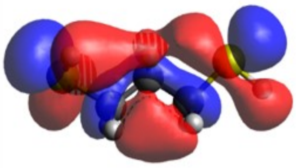<br>-0.26	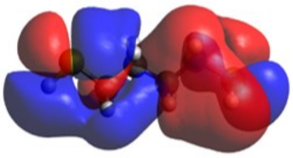<br>-0.45	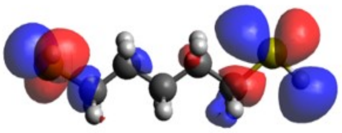<br>-0.49

Table S8. Comparison between the frontier molecular orbitals of alkane chain, including *n* =6, 7 and 8 linear chains in the gas phase (thiol terminal group).

**Chains**	**6**	**7**	**8**
**Mol.**<br><br>**E_F (eV)**	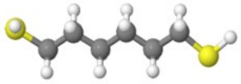<br>-2.40	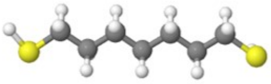<br>-2.0	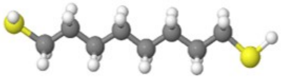<br>-1.7
**HOMO**<br><br>**eV**	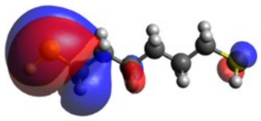<br>-4.75	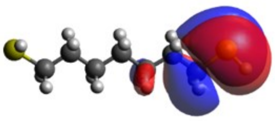<br>-4.70	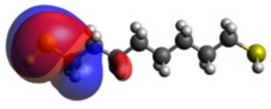<br>-4.72
**LUMO**<br><br>**eV**	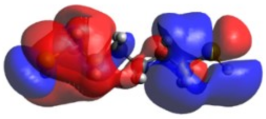<br>-0.50	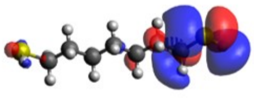<br>-0.54	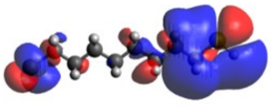<br>-0.56

Table S9. Comparison between the frontier molecular orbitals of alkane chain, including *n* =9 and 10 linear chains in the gas phase (thiol terminal group).

Chains	9	10
Mol.<br><br>E_F (eV)	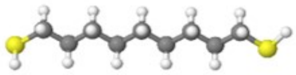<br>-1.5	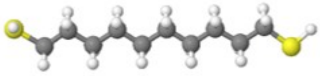<br>-1.2
HOMO<br><br>eV	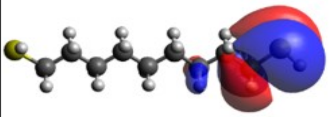<br>-4.7	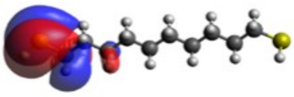<br>-4.71
LUMO<br><br>eV	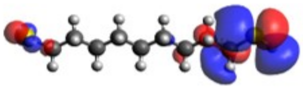<br>-0.56	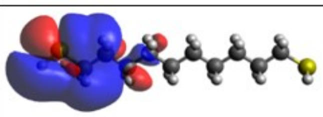<br>-0.57

Table S10. Comparison between the frontier molecular orbitals of alkane chain, including *n* =3, 4 and 5 linear chains in the gas phase (direct carbon anchor).

Chains	3	4	5
Mol.<br><br>E_F (eV)	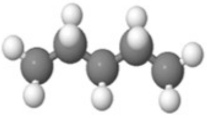<br>-6.0	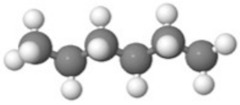<br>-5.6	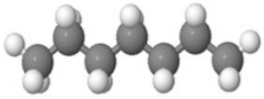<br>-5.4
HOMO<br><br>eV	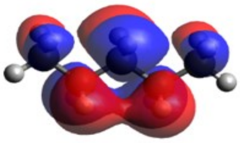<br>-7.2	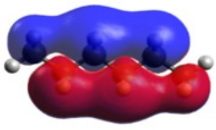<br>-7.19	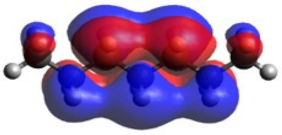<br>-7.2
LUMO<br><br>eV	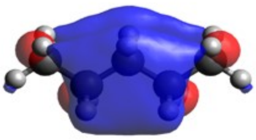<br>-2.1	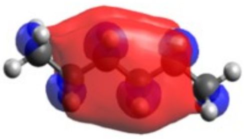<br>-2.1	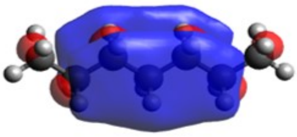<br>-2.0

Table S11. Comparison between the frontier molecular orbitals of alkane chain, including *n* =6, 7 and 8 linear chains in the gas phase (direct carbon anchor).

**Chains**	6	7	8
**Mol.**<br><br>**E_F (eV)**	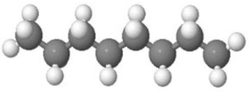<br>-5.20	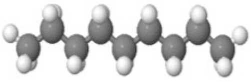<br>-5.1	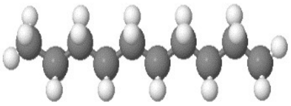<br>-4.9
**HOMO**<br><br>**eV**	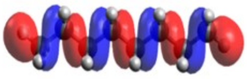<br>-7.14	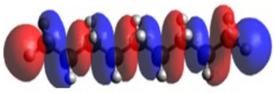<br>-7.1	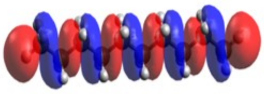<br>-7.0
**LUMO**<br><br>**eV**	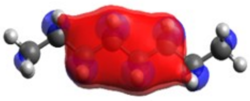<br>-2.0	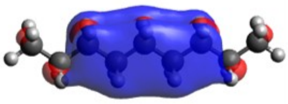<br>-2.1	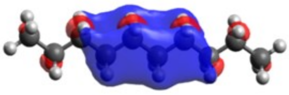<br>-2.0

Table S12. Comparison between the frontier molecular orbitals of alkane chain, including *n* =9 and 10 linear chains in the gas phase (direct carbon anchor).

**Chains**	9	10
**Mol.**<br><br>**E_F (eV)**	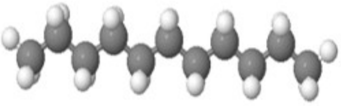<br>-4.8	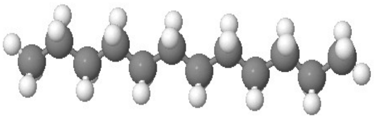<br>-4.7
**HOMO**<br><br>**eV**	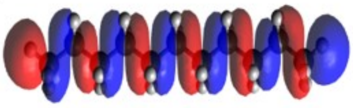<br>-7.0	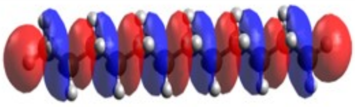<br>-6.9
**LUMO**<br><br>**eV**	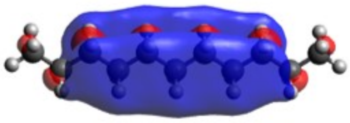<br>-1.98	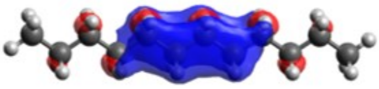<br>-1.9

3. Binding energy of four terminal groups on gold

To calculate the optimum binding distance for the alkane linear chains with difference terminal groups (*Au-NH₂, Au-SMe, Au-S* and *Au-C*) binding to the gold (111) surfaces, DFT and the counterpoise method were used, which removes basis set superposition errors (BSSE). The binding distance was defined as the distance between the gold surface and the terminated end group/atom of the molecule. The ground state energy of the total system was calculated using SIESTA and is denoted E_{AB}^{AB}, with the parameters defined in the main text. Here the gold leads consist of 6 layers of 30 atoms. The energy of each monomer was then calculated in a fixed basis, which is achieved through the use of ghost atoms in SIESTA. Hence the energy of the individual molecule in the presence of the fixed basis is defined as E_{A}^{AB} and for the isolated gold as E_{B}^{AB}. The binding energy is then calculated using the following equation: [4-5]

$$Binding\ Energy = E_{AB}^{AB} - E_{A}^{AB} - E_{B}^{AB} \qquad \text{(S1)}$$

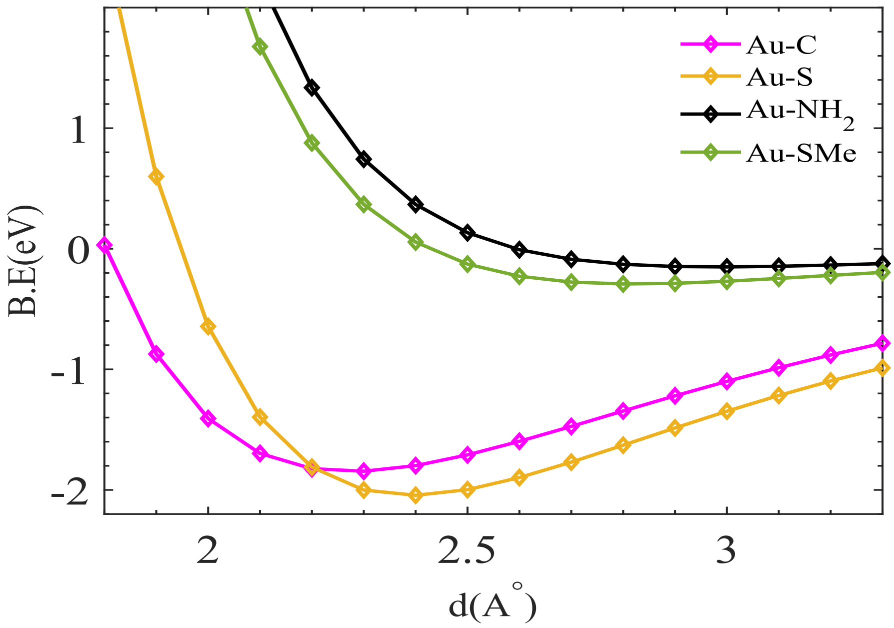

Supplementary Figure 2. Binding energy of alkane linear chains to gold as a function of molecule-contact distance. The equilibrium distance (i.e. the minimum of the binding energy curve) is found to be approximately 2.3, 2.4, 2.8 and 3.0 Å, for *Au-C, Au-S, Au- NH_2* and *Au-SMe* (top to bottom).

4. Optimised DFT Structures of Compounds in their Junctions

After calculating the optimum geometries of the isolated molecular chains, the hydrogen atoms were removed from the terminal thiol and direct carbon groups and the chains were attached to gold electrodes. Finally, the geometries of the whole junctions were further relaxed. Here are a few examples of optimised DFT structures of the alkane chains, in their Au junctions as shown in Supplementary Figure 3.

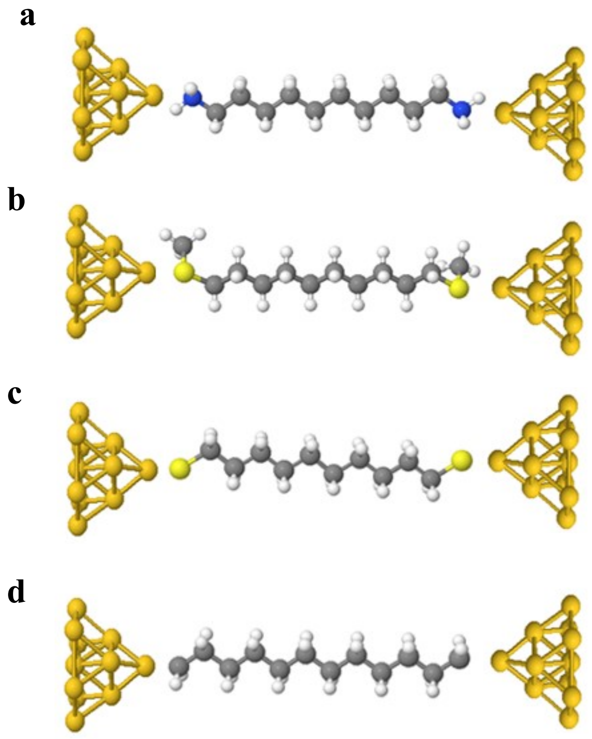

Supplementary Figure 3. Examples of fully relaxed alkane derivatives in Au|alkane chains|Au junctions: (a), (b), (c) and (d): n =10 linear chains connected to gold electrodes via amine, thiomethyl, thiol and direct carbon anchor groups respectively (for clarity only 1 chain out of 8 are shown for each terminal group).

To calculate the electrical transport through Au|alkane chains|Au junctions, we employed GOLLUM code. Gollum is a quantum transport multi-functional code that evaluates the charge, spin and electronic contribution to the thermal transport properties of multi-terminal junctions. In contrast to NEGF (Nonequilibrium Green's function), quantum transport codes, GOLLUM is based on equilibrium transport theory. GOLLUM code starts from the Hamiltonian that describes a system and calculates the quantum-mechanical scattering matrix S, from which a wide range of measurable quantities can be predicted (for more detail see ref.1).

5. Conductance comparison between linear chains of different terminal groups

In this section, we compare the conductance *G* of linear chains of the four different terminal groups, including amine *Au-NH₂*, thiomethyl *Au-SMe*, thiols *Au-S* and direct carbon *Au-C*. Supplementary Figures 3-7 show that the DFT simulations predict that the conductances follow the order *C* > *S* > *SMe* > *NH₂*. In other words, (*Au-C*) conductance values were the highest, while (*Au-Amine*) yielded the lowest conductance values.

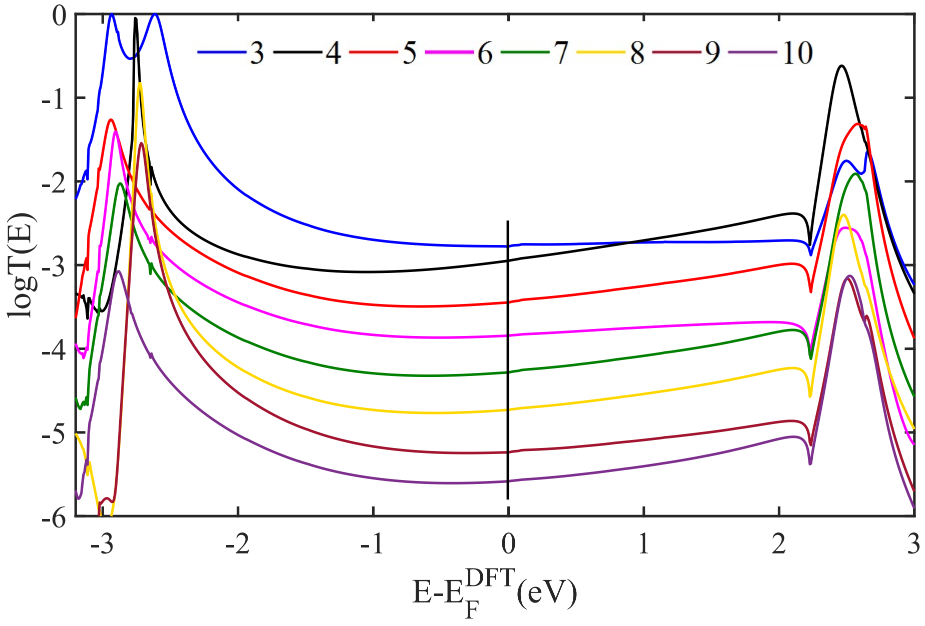

Supplementary Figure 4. Transmission coefficient curves of alkane chains with (*Au-Amine*), for *n*=3 to 10, carbon atoms of linear chains against electron energy *E*.

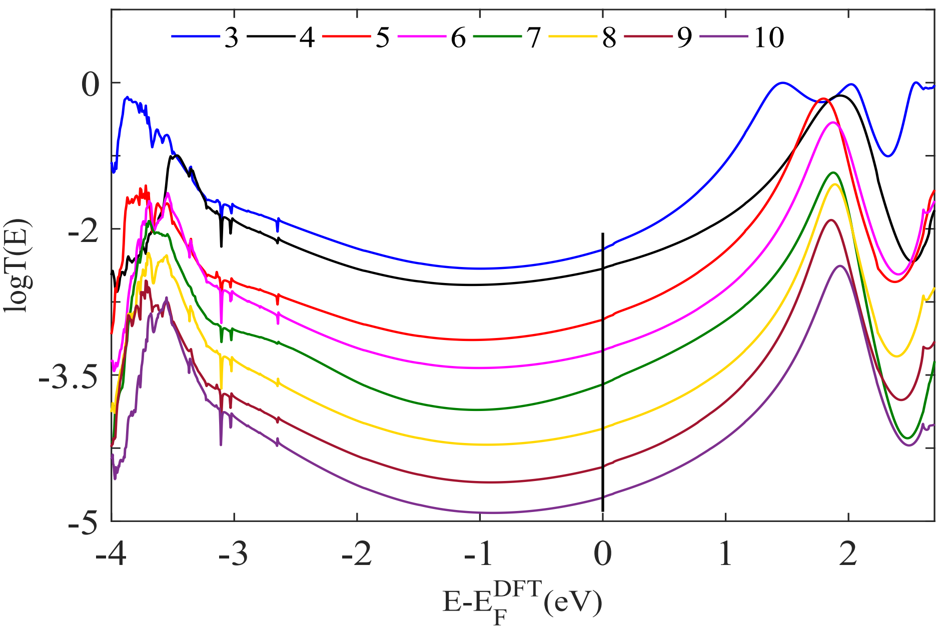
3
4
5
6
7
8
9
10
0
-2
-3.5
-5
logT(E)
-4
-3
-2
-1
0
1
2
E-E$_F^{DFT}$(eV)

Supplementary Figure 5. Transmission coefficient curves of alkane chains with (*Au-SMe*), for *n*=3 to 10, carbon atoms of linear chains against electron energy *E*.

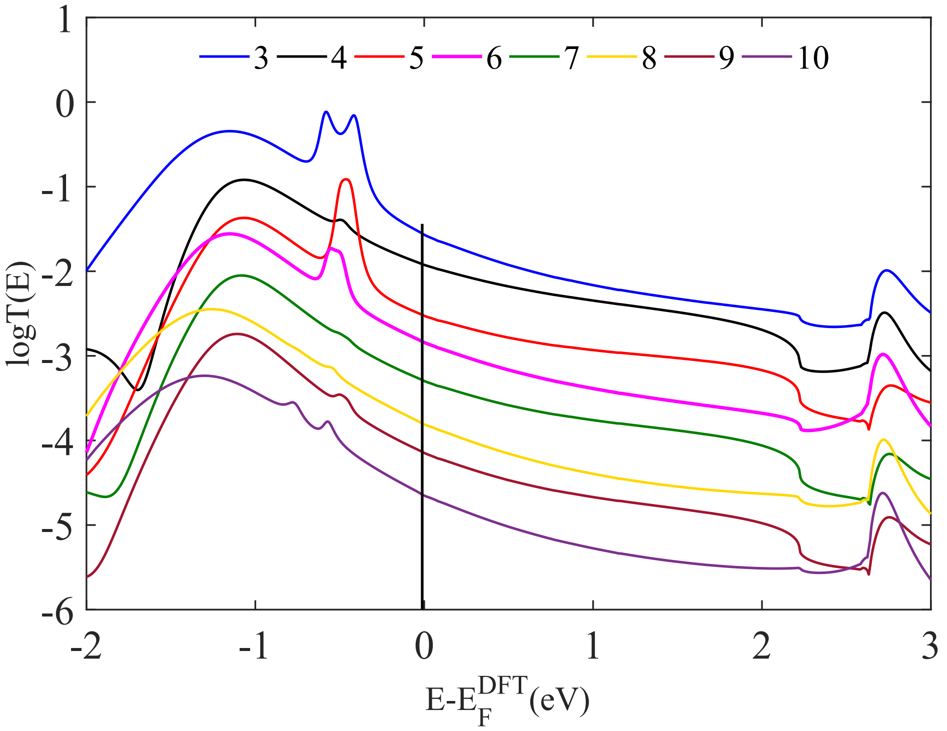

Supplementary Figure 6. Transmission coefficients of alkanes chains with (*Au-S*), for *n*=3 to 10, carbon atoms of linear chains against electron energy *E*.

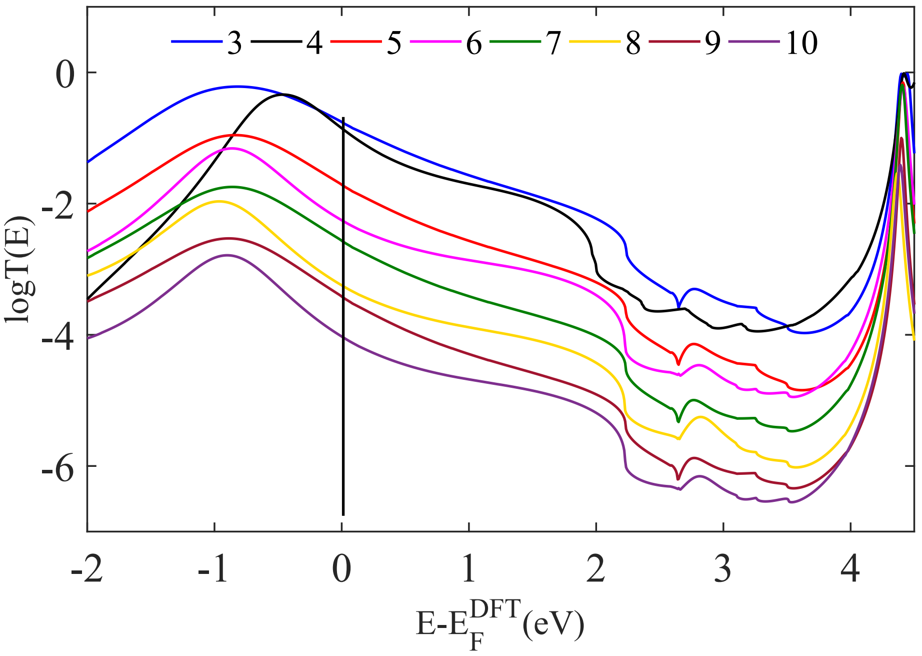

3
4
5
6
7
8
9
10
0
-2
-4
-6
logT(E)
-2
-1
0
1
2
3
4
E-E$_F^{DFT}$(eV)

Supplementary Figure 7. Transmission coefficients of alkanes chains with (*Au-C*), for *n*=3 to 10, carbon atoms of linear chains against electron energy *E.*

References

1. J. M. Soler, E. Artacho, J. D. Gale, A. García, J. Junquera, P. Ordejón and D. J. J. o. P. C. M. Sánchez-Portal, *J. Phys.: Condens. Matter*, 2002, **14**, 2745.
2. A. Emilio, E. Anglada, O. Diéguez, J. D. Gale, A. García, J. Junquera, R. M. Martin, P. Ordejón, J. M. Pruneda, D. Sánchez-Portal and J. M. Soler, *J. Phys.: Condens. Matter*, 2008, **20**, 064208.
3. Kobko, N.; Dannenberg, J., Effect of basis set superposition error (BSSE) upon ab initio calculations of organic transition states. *The Journal of Physical Chemistry A* 2001, *105* (10), 1944-1950.
4. Sherrill, C. D., Counterpoise correction and basis set superposition error. *School of Chemistry and Biochemistry, Georgia Institute of Technology* 2010.
5. Sinnokrot, M. O.; Valeev, E. F.; Sherrill, C. D., Estimates of the ab initio limit for π− π interactions: The benzene dimer. *Journal of the American Chemical Society* 2002, *124* (36), 10887-10893.
